# Factors Associated with Dental Plaque, Gingivitis, and Caries in a Pediatric Population: A Records-Based Cross-Sectional Study

**DOI:** 10.3390/ijerph17228595

**Published:** 2020-11-19

**Authors:** Esti Davidovich, Julie Grender, Avi Zini

**Affiliations:** 1 Department of Pediatric Dentistry, Faculty of Dental Medicine, Hebrew University & Hadassah, Jerusalem 91120, Israel; esti@dr-st.co.il; 2 Statistics and Data Management, The Procter & Gamble Company, Mason, OH 45040, USA; grender.jm@pg.com

**Keywords:** caries, children, electric toothbrush, gingivitis, logistic model, manual toothbrush, oscillating-rotating toothbrush, plaque, paediatric, retrospective chart review

## Abstract

This retrospective, cross-sectional study evaluated dental records of 1000 healthy children to determine factors associated with plaque, gingivitis, and caries. A logistic model for plaque and gingivitis (mild versus moderate/severe) and caries (yes/no) was carried out separately for each variable using the following potential factors: Age, Gender, Brush Type, Starting Age of Brushing, Brushing Frequency, and Bite Type. Data from 998 children (median age: 4 years, 10 months (range: 2.5–7 years)) were analyzed. Sixty-four percent were manual toothbrush users; 36% were oscillating-rotating electric toothbrush users. For plaque and gingivitis, but not caries, Brush Type was more impactful than Brushing Frequency. Age influenced the severity of plaque and gingivitis, with increases in the odds of having moderate/severe plaque or gingivitis associated with increasing age. The probability of caries increased until approximately age 5 and then decreased until age 7. Oscillating-rotating brush users were more likely to present with less plaque, gingivitis, and caries, with 6.0, 5.1, and 1.4 times greater odds of having mild (versus moderate/severe) plaque, less severe gingivitis, and being caries-free, respectively, than manual brush users. Similarly, brushing twice daily and starting brushing at an earlier age were associated with better oral health outcomes. Children with anterior bite abnormalities had increased odds of developing moderate/severe plaque and gingivitis than children with normal anterior bites. Gender was not a statistically significant factor associated with plaque, gingivitis, or caries. Children’s oral health is influenced by toothbrush type, starting age of brushing, compliance with twice-daily brushing, and bite abnormalities.

## 1. Introduction

Childhood caries remains a significant health problem globally, with an estimated worldwide incidence of 1.76 billion among children with deciduous teeth. [1] Consumption of sugars, the most significant dietary factor in the development of caries, has tripled worldwide over the past 50 years and is expected to continue to increase [2,3]. Children frequently consume excess added sugars in the form of snacks, processed foods, and sugar-sweetened beverages [4,5]. The majority of dietary sugars are metabolized by cariogenic bacteria in dental plaque to form acids, which then demineralize the enamel [6,7].

Proper oral hygiene, primarily through effective toothbrushing, reduces plaque, and is critical for prevention of caries [8]. Plaque control also reduces gingivitis, which in turn helps to prevent the development of periodontitis later in life [9]. Good oral hygiene habits formed at an early age thus help to establish a foundation for continued oral health in adulthood. Toothbrushing in children, however, is a complex behavior influenced by a number of developmental and social factors, including limited dexterity, as well as difficulty maintaining routines that pose barriers to effective implementation [10,11].

Abundant evidence from randomized clinical studies and systematic reviews in adult populations demonstrates that electric toothbrushes provide superior plaque removal [12,13,14,15] and gingivitis reduction benefits [13,15,16] compared with manual brushes. There is strong evidence in adults for the use of oscillating-rotating electric toothbrushes in particular, which have been shown to reduce more plaque and to improve gingival health compared with both manual [12,15] and sonic electric brushes [14,15,17]. While clinical studies of toothbrushing in children are limited, a recent systematic review and meta-analysis showed that electric toothbrushes were also more effective at removing plaque than manual toothbrushes in pediatric populations [18]. A fuller understanding of the potential benefits of electric toothbrush use in children, as well as the possible impact of additional important factors on oral health early in life, such as age, bite type, and brushing frequency, is warranted.

Given the lack of a comprehensive investigation focused on toothbrushing in young children and the potential for multiple developmental and behavioral factors to influence oral health outcomes, an extensive records review was undertaken as a retrospective assessment of factors related to brushing regimen with the objective of determining key factors, including Age, Gender, Brush Type, Starting Age of Brushing, Brushing Frequency, and Bite Type, associated with plaque, gingivitis, and caries in a pediatric population aged 2.5 to 7 years.

## 2. Materials and Methods

In this retrospective, cross-sectional study, patient records of 1000 healthy children aged 2.5 to 7 years who visited a pediatric dental clinic in Tel Aviv, Israel from April to June 2019 and met all inclusion criteria, including having complete records, were reviewed by a trained and experienced specialist in pediatric dentistry [19,20]. The sample was limited to 1000 children prior to the study. Children with existing systemic diseases that required any medication or regular medical treatment were excluded, with the exception of Attention-Deficit/Hyperactivity Disorder, which was not excluded. Every child was included in the study only once. The study was approved by the Ethics Committee for Human Studies of the Hebrew University, Jerusalem #08082019. The following variables related to brushing regimen were collected for each subject: age (months), gender, brush used at home (manual, electric), age brushing started (months; by caregiver)), supervised brushing (yes/no), daily brushing frequency (once, twice), dentition (primary, mixed), anterior bite (normal, open, cross), currently using pacifier (yes, no), current thumb sucker (yes, no), plaque (mild, moderate, severe), gingivitis (mild, moderate, severe), caries (yes/no). Since thumb sucking and pacifier use are confounded with the child’s age, these factors could not be considered for the statistical model.

A logistic model for plaque according to Modified Turesky Plaque index [21,22], mild (no plaque or isolated areas of plaque at gingival margin) versus moderate (thin plaque covering up to 1/3 of tooth surface) /severe (plaque covering more than 1/3 of tooth surface), gingivitis according to Modified Gingival Index [23], mild (normal or slight change in color, little change in the texture of any portion of but not the entire marginal or papillary gingival unit) versus moderate (mild inflammation; criteria as above but involving the entire marginal or papillary gingival unit or mild inflammation; criteria as above but involving the entire marginal or papillary gingival unit) /severe inflammation) and caries experience (yes/no; including untreated caries and fillings; white spot lesions were not included) was carried out separately for each variable using the following potential factors: Age, Gender, Brush Type, Starting Age of Brushing, Brushing Frequency, and Bite Type. The strategy was to identify a group of variables that were reasonably not confounded as potential candidate factors for the model. A statistical model was fit to the data where linear effects, quadratic effects, and all two-way interactions were assessed. Backward Stepwise Regression was utilized to determine a preliminary model, which was refined with other methods, and included inspection of R^2^, mean squared error and residual plots. Main effects (linear effects or quadratic effects) that were significant at the 0.05 level or interaction terms that were significant at the 0.1 level were retained in the model. Since there were only 2 sonic brush users, the statistical modelling analysis was undertaken on records from 998 children: 635 manual toothbrush users and 363 electric, rechargeable oscillating-rotating toothbrush users.

## 3. Results

### 3.1. Subject Characteristics and Demographics

**Figure 1:**
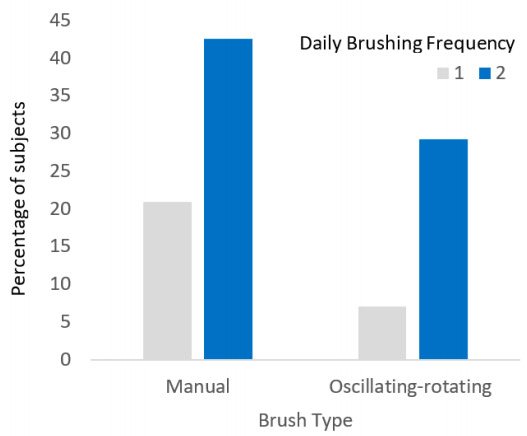
Distribution of Daily Brushing Frequency by Brush Type.

Data from the dental records of 1000 children aged 2.5 to 7 years were reviewed and recorded. The median age of children was 4 years and 10 months (range, 2.5 to 7.0 years). Approximately half (53.3%) were female, 83.9% had primary dentition, and 73.6% had a normal bite. The majority (63.5%) of children were manual toothbrush users, 36.3% were electric-rechargeable oscillating-rotating toothbrush users, and <1% (n = 2) were electric-rechargeable sonic toothbrush users. Seventy-two percent of children brushed their teeth twice a day. The median age that children started to use a toothbrush was 1 year (range, 0.5 to 2.0 years). Approximately three-quarters (74.7%) had their brushing supervised (Table 1). The distribution of daily brushing frequency by brush type is reported in Figure 1, which shows a similar difference between once-a-day and twice-a-day brushing for the manual and oscillating-rotating brushers.

### 3.2. Dental Plaque Modeling

Brush Type, Brushing Frequency, Age, Starting Age of Brushing, Bite Type, and Starting Age of Brushing by Bite Type interaction were statistically significant (*p* < 0.02) factors associated with severity of plaque; Brush Type was the most impactful factor associated with plaque severity (Table 2). Oscillating- rotating brush users were more likely to present with less plaque than manual brush users, with the odds of having mild (versus moderate/severe) plaque with an oscillating-rotating brush being 6.0 times greater than with a manual brush (odds ratio: 6.03 (95% CI: 4.40, 8.27), *p* < 0.001). For Brushing Frequency, the odds of having mild (versus moderate/severe) plaque with twice-daily brushing were 4.4 times greater compared to once-daily brushing (odds ratio: 4.36 (95% CI: 3.04, 6.25); *p* < 0.001) (Table 2). Age also contributed to plaque severity, with a 3% increase in the odds of having moderate/severe plaque for every 1-month increase in age. At the average brushing starting age of 11.2 months, children with normal anterior bites had 1.7 times the odds of developing mild (versus moderate/severe) plaque compared to children with anterior bite abnormalities (odds ratio: 1.68 (95% CI, 1.12, 2.52); *p* = 0.012) (Table 2).

**Figure 2:**
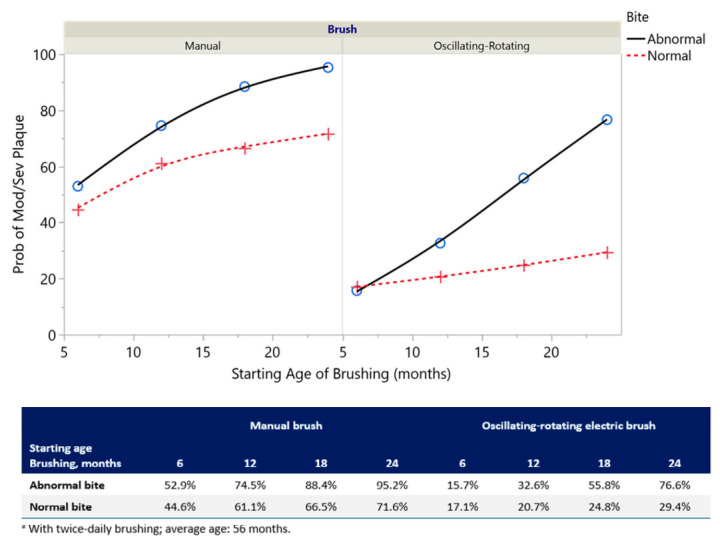
Probability ^a^ of moderate/severe plaque by Brush Type, Bite Type, and Starting Age of Brushing (months) from the model in Table 2.

The probability of having moderate/severe plaque based on Bite Type (normal/abnormal) and Starting Age of Brushing for both oscillating-rotating electric and manual brush users is shown in Figure 2. The interaction between the type of Anterior Bite and Starting Age for Brushing impacted the odds ratios for severity of plaque accumulation; specifically, the impact of a child’s bite type on plaque level increased as the child delayed in starting a brushing routine. Here a 12.8% increase in the odds ratio was observed for every 1-month delay in the start of brushing. Thus, the children with abnormal anterior bites who did not start brushing until 18 months of age showed 3.8 times greater odds of presenting with moderate/severe (versus mild) plaque than those children with normal anterior bites. Among children with normal anterior bites who started brushing at 18 months of age, oscillating-rotating electric brush users had a 24.8% probability of developing moderate/severe plaque, while manual brush users had a 66.5% probability. Probabilities of developing moderate/severe plaque for children with anterior bite abnormalities who started brushing at 18 months of age were 55.8% and 88.4% for oscillating-rotating electric and manual brush users, respectively (Figure 2).

### 3.3. Gingivitis Modeling

Brush Type, Brushing Frequency, Age, Starting Age of Brushing, and Bite were statistically significant (*p* ≤ 0.006) factors associated with severity of gingivitis; Brush Type was the most influential factor associated with gingivitis severity (Table 3). Oscillating-Rotating brush users were more likely to have milder signs of gingivitis, with manual brush users having 5.1 times greater odds of presenting with moderate/severe (versus mild) gingivitis than oscillating-rotating brush users (odds ratio: 5.12 (95% CI: 3.41, 7.69), *p* < 0.001). For Brushing Frequency, the odds of having mild (versus moderate/severe) gingivitis with twice-daily brushing were 3.1 times higher when compared to once-daily brushing (odds ratio: 3.08 (95% CI: 2.22, 4.28); *p* < 0.001) (Table 3). Age also contributed to gingivitis severity, with a 4% increase in the odds of having moderate/severe gingivitis for every 1-month increase in the child’s age. For every 1-month delay in the start of brushing, there was a 5% increase in the odds of having moderate/severe gingivitis. Additionally, children with abnormal anterior bites had 1.8 times greater odds of developing moderate/severe (versus mild) gingivitis compared to children with normal anterior bites (odds ratio: 1.75 (95% CI, 1.17, 2.60); *p* = 0.006) (Table 3).

**Figure 3:**
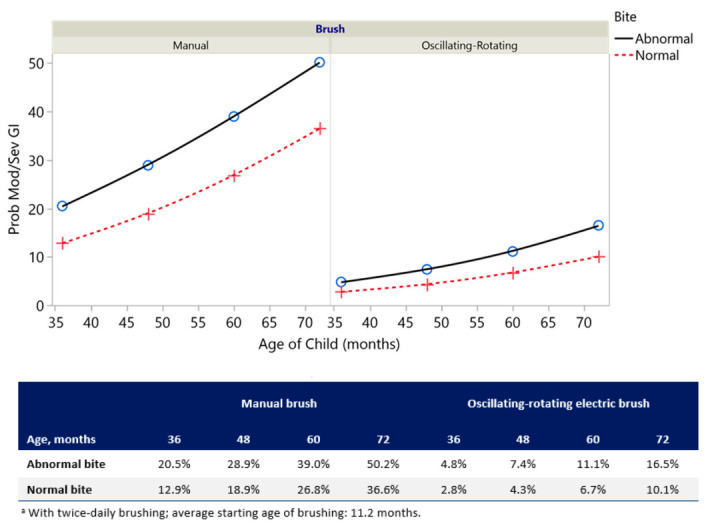
Probability ^a^ of moderate/severe gingivitis by Brush Type, Bite Type, and Age (months) from the model in Table 3.

The probability of having moderate/severe gingivitis based on Bite Type and Age for both oscillating-rotating electric and manual brush users is shown in Figure 3. The probability of having moderate/severe gingivitis was lower with oscillating-rotating electric brush use than with manual brush use among children with either normal or abnormal anterior bites. Oscillating-rotating electric brush users with normal anterior bites had the lowest probability of developing moderate/severe gingivitis at each age (Figure 3).

### 3.4. Caries Modeling

**Figure 4:**
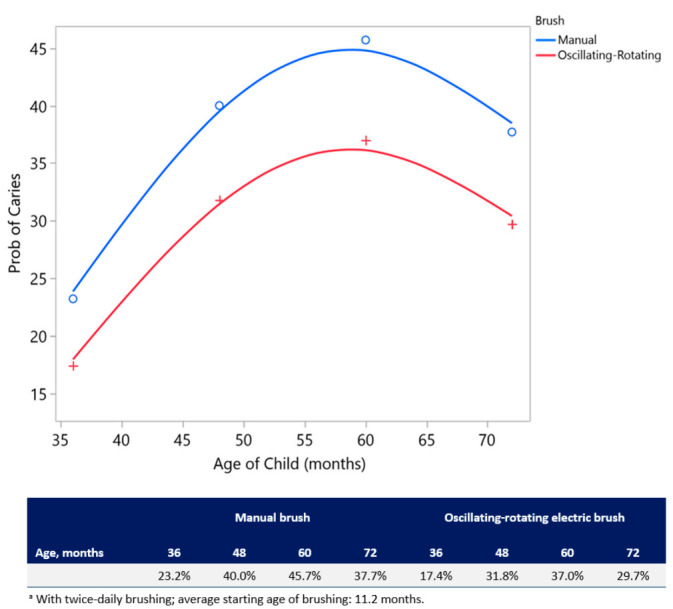
Probability ^a^ of having caries by Brush Type and Age (months) from the model in Table 4.

Brush Type, Brushing Frequency, Age, Age × Age (quadratic contribution of age), and Starting Age of Brushing were statistically significant (*p* < 0.05) factors associated with the presence of caries; Brushing Frequency was the most impactful factor for caries (Table 4). The odds of being caries-free with twice-daily brushing were 1.7 times greater than with once-daily brushing (odds ratio: 1.67 (95% CI: 1.25, 2.24); *p* < 0.001) (Table 4). For Brush Type, the odds of being caries-free using an oscillating-rotating electric brush were 1.4 times greater than using a manual brush (odds ratio: 1.43 (95% CI: 1.08, 1.91), *p* = 0.013). Age had a quadratic effect on the incidence of caries, where the probability of having caries increased until approximately 60 months (5 years) of age and then decreased until age 7 (Figure 4). For every 1-month delay in the start of brushing, there was a 5% increase in the odds of having caries.

## 4. Discussion

This retrospective, cross-sectional study involving the review of dental records demonstrates that oral health in children aged 2.5 to 7 years is influenced significantly by type of toothbrush, the starting age for tooth brushing, compliance with twice-daily brushing, and bite abnormalities. Brush Type was the factor having the greatest association with the severity of plaque and gingivitis, with oscillating-rotating electric toothbrush use significantly increasing the likelihood of presenting with lower levels of plaque and gingivitis (mild plaque and mild gingivitis versus moderate/severe) compared to manual brush use. Similarly, brushing twice daily was associated with better oral hygiene, with significantly increased odds of developing mild plaque and mild gingivitis (versus moderate/severe plaque and gingivitis) with twice-daily brushing compared to once-daily brushing. Age influenced the severity of plaque and gingivitis, with increases in the odds of having moderate or severe plaque and gingivitis for every 1-month increase in age. Children with anterior bite abnormalities had significantly higher odds of developing moderate/severe (versus mild) plaque and gingivitis compared to children with normal anterior bites. Regardless of the age when brushing begins, children with abnormal anterior bites who used oscillating-rotating electric toothbrushes were less likely to develop moderate/severe plaque or gingivitis than children with normal bites who used manual brushes; these findings suggest that oscillating-rotating electric toothbrush use helps overcome some of the barriers to optimal oral health associated with having a cross-bite or open bite.

The importance of toothbrushing in reducing plaque, gingivitis, and caries in children is well-documented [24,25,26]; however, few studies differentiate with regard to the type of toothbrush that might be most beneficial for pediatric populations. While toothbrushing research among children is limited, the findings of this study are consistent with published clinical studies and meta-analyses demonstrating the superior plaque removal efficacy of electric toothbrushes versus manual brushes in young children [18,19,20]. Our findings, which are strongly in favor of the oscillating-rotating electric toothbrush, suggest the importance of brush technology type is key to maintaining and improving oral hygiene, managing plaque, and preventing gingivitis. Starting Age of Brushing was a significant factor associated with the severity of plaque, gingivitis, and the presence of caries, with increased odds of poor oral hygiene outcomes for every 1-month delay at the start of brushing. Other studies in the literature also show an association between plaque/caries with a later starting age of brushing. Refs. [27,28] taken together, these results underscore the importance of starting toothbrushing at an early age and establishing a twice-daily brushing routine using an electric toothbrush.

The American Academy of Pediatric Dentistry guidelines explain clearly that plaque accumulation is strongly associated with caries development in young children, and is valuable in assessing risk, especially in pre-school children [29]. Habits are formed early in life, with oral hygiene practices adopted in childhood tracking through adolescence and into adulthood [30,31]. Thus, a good toothbrushing routine instilled at an early age should establish trajectories into adulthood that improve the chances of a healthy periodontium and reduce the risk of caries. Unfortunately, many parents are not fully equipped to establish proper oral hygiene for their children [32], so educational programs have been developed at school and elsewhere to assist parents in this role. For example, work has been conducted at the University of Leeds to develop standardized oral health training and resource interventions (Health Visitors delivering Advice in Britain on Infant Toothbrushing [HABIT] and “Strong Teeth”) to improve parental supervised brushing in infants and young children [10,33]. The findings of our study, demonstrating the influence of multiple variables on oral health in children, reinforce the complexity of oral hygiene behaviors and support the use of such standardized interventions.

Chart reviews offer advantages over clinical studies in that they are easier to manage logistically, require fewer resources, and offer a real-world perspective with no study influence or concern of novelty effects [34]. In addition, patient compliance, of particular concern in studies of young children, is not a factor in chart review studies. There are, however, some limitations to this research. While retrospective chart reviews provide important complementary information, they lack the robustness of randomized clinical studies. The findings described here indicate associations, and we are unable to make statements about causation. The results are also based on records from a single dental clinic and therefore may not be representative of the pediatric population at-large. We cannot assess how long children were using the type of brush noted, which could affect our results. Possible biases also should be taken into consideration, such as the issue of self-reported data regarding health behaviors. This is most likely a factor for the brushing frequency variable, as parents may not report accurately brushing behaviors that are inconsistent with the child’s dentist’s recommendation. Finally, the effect of toothbrush type was most substantial for plaque and gingivitis, while daily brushing frequency was more impactful for caries. Caries was a “yes/no” categorization in this study, with no decayed, missing, and filled surfaces (DMFS) or International Caries Diagnosis and Assessment System (ICDAS) exam values, thus limiting the conclusions that can be drawn on factors associated with caries [15,16]. Notably, a recent 11-year cohort study assessing electric versus manual brush use in adults found that electric toothbrush users had 17.7% lower DMFS progression throughout the study period [35].

## 5. Conclusions

Children’s oral health is influenced significantly by type of toothbrush, the starting age for tooth brushing, compliance with twice-daily brushing, and bite abnormalities. The variables evaluated in this study related to brushing regimen are well-recognized in dental practice and oral research. These findings can be used to support dental professional recommendations on early uptake of tooth brushing and the use of electric toothbrushes, specifically oscillating-rotating toothbrushes, which comprised almost the entirety of the electric brush subject cohort of this study.

## Figures and Tables

**Table 1:** Subject demographics and characteristics.

Characteristic	(N = 1000)
**Age, years**	
Mean	4.7
Median (range)	4.8 (2.5 to 7.0)
**Gender, %**	
Female	53.3
Male	46.7
**Dentition, %**	
Primary	83.9
Mixed	16.1
**Bite, %**	
Normal	73.6
Open bite	25.9
Cross-bite	0.5
**Brush type, %**	
Manual	63.5
Oscillating-rotating electric	36.3
Sonic electric	0.2
**Brushing frequency, %**	
Twice-daily	72.0
Morning only	8.8
Evening only	19.2
**Age at start of tooth brushing, months**	
Mean	11.2
Median (range)	12.0 (6.0 to 24.0)
**Supervised brushing, %**	74.7
**Pacifier use, % ^a^**	21.5
**Thumb sucker, % ^a^**	0.2
**Plaque severity, %**	
Mild	45.0
Moderate	47.5
Severe	7.5
**Gingivitis severity, %**	
Mild	73.6
Moderate	23.4
Severe	3.0
**Caries present, %**	38.8

^a^ At the time the questionnaire was administered.

**Table 2:** Factors contributing to the plaque model.

Factor ^a^	*p*-Value	Odds Ratio (95% CI) ^b^
Brush Type (oscillating—rotating/manual)	<0.001	6.03 (4.40, 8.27)
Brushing Frequency (2×/1×)	<0.001	4.36 (3.04, 6.25)
Bite Type (normal/abnormal) ^c^	0.012	1.68 (1.12, 2.52)
Age (months)	<0.001	1.03 (1.02, 1.04)
Starting Age of Brushing (months)	<0.001	NA since Starting Age of Brushing × Bite Type in model
Starting Age of Brushing × Bite	0.012	

NA: not applicable; ^a^ For the three categorical variables, the reference categories are manual brush, 1×/day brushing, and abnormal bite. ^b^ Odds ratios represent the odds of having mild plaque versus moderate/severe plaque after adjusting for all other variables. ^c^ At average starting age of brushing of 11.2 months.

**Table 3:** Factors contributing to the gingivitis model.

Factor ^a^	*p*-Value	Odds Ratio (95% CI) ^b^
Brush type (oscillating-rotating/manual)	<0.001	5.12 (3.41, 7.69)
Brushing frequency (2×/1×)	<0.001	3.08 (2.22, 4.28)
Bite (normal/abnormal)	0.006	1.75 (1.17, 2.60)
Starting Age of brushing (months)	0.001	1.05 (1.02, 1.08)
Age (months)	<0.001	1.04 (1.02, 1.05)

^a^ For the three categorical variables, the reference categories are manual brush, 1×/day brushing, and abnormal bite. ^b^ Odds ratios represent the odds of having mild gingivitis *versus* moderate/severe gingivitis after adjusting for all other variables.

**Table 4:** Factors contributing to the caries model.

Factor ^a^	*p*-Value	Odds Ratio (95% CI) ^b^
Brushing frequency (2×/1×)	<0.001	1.67 (1.25, 2.24)
Brush type (oscillating-rotating/manual)	0.013	1.43 (1.08, 1.91)
Starting Age of brushing (months)	<0.001	1.05 (1.02, 1.07)
Age (months)	0.045	NA since age × age in model
Age × Age (months)	<0.001	

NA: not applicable; ^a^ For the two categorical variables, the reference categories are 1×/day and manual brush. ^b^ Odds ratios represent the odds of not having caries *versus* having caries after adjusting for all other variables.
